# Retraction Note: Astrocyte-derived MMP-9 is a key mediator of pseudorabies virus penetration of the blood–brain barrier and tight junction disruption

**DOI:** 10.1186/s13567-026-01826-7

**Published:** 2026-08-24

**Authors:** Ying Zhang, Xianghua Shu, Ying Zhang, Chunlian Song, Yi Wu, Kesi Cui, Xue Zhang, Yalong Sun, Hong Shen, Qianfei Wei, Jianqin Li, Yue Shu

**Affiliations:** 1 Present Address: College of Veterinary Medicine of Yunnan Agricultural University, Kunming, 650201 Yunnan Province China; 2 The Faculty of Science and Mathematics, Auburn University, Auburn, AL USA

**Retraction Note** **:** **Veterinary Research (2025) 56:72** 10.1186/s13567-025-01486-z

The Editor-in-Chief has retracted this article. Following publication, the corresponding author notified the journal regarding overlap between this article and a previously published article in Chinese with overlapping authors [1]. The Editor-in-Chief determined that this article is therefore redundant.

The authors have not responded to correspondence regarding this retraction notice.
